# One-Step Hybrid Bending/Diffusion Bonding Process and Analysis of the Bonding Characteristics of Titanium Alloy Sheets

**DOI:** 10.3390/ma16134516

**Published:** 2023-06-21

**Authors:** Kang Hyun Kim, Hyeonil Park, Dong Jun Lee, Yong Nam Kwon, Namhyun Kang, Jong-Hwa Hong

**Affiliations:** 1 Aerospace Materials Center, Korea Institute of Materials Science, 797 Changwon-daero, Changwon 51508, Republic of Korea; sgngkim517@kims.re.kr (K.H.K.); hipark@kims.re.kr (H.P.); djlee@kims.re.kr (D.J.L.); kyn1740@kims.re.kr (Y.N.K.); 2 Department of Materials Science and Engineering, Pusan National University, 2 Busandaehak-ro 63beon-gil, Busan 46241, Republic of Korea

**Keywords:** one-step hybrid bending/diffusion bonding process, titanium alloy sheets, numerical simulation, viscoplasticity, aircraft parts

## Abstract

A one-step hybrid bending/diffusion bonding process (HB/DBP) was developed for application to Ti-6Al-4V sheets to effectively improve buy-to-fly (BTF) ratio of aircraft parts, integrating sequential diffusion bonding followed by a bending process. The resulting bonding characteristics of these titanium alloy sheets were analyzed. Microstructural analysis and mechanical lap shear tests were performed to estimate the bonding quality. Additionally, bonding ratio, thickness strain, and shear strength were evaluated in relation to pressure under increasing temperature. When the applied pressure was lower than 0.5 MPa, early failure occurred at the joint of the specimens. However, when high pressure was applied, early failure occurred near the joint. To discuss the phenomenon, time-dependent viscoplastic material properties were characterized, and a numerical simulation analysis was performed. Viscoplastic deformation was observed around the bending area, which caused weakness around the bond under high-pressure conditions. A prototype of a Y-shaped heat shield was manufactured and the buy-to-fly ratio was effectively improved using the newly developed process. This study demonstrates the potential of applying the developed process for producing aircraft parts and the importance of viscoplastic behavior for the analysis of final product reliability.

## 1. Introduction

Titanium has excellent corrosion resistance, creep, and fatigue properties, and a high strength-to-weight ratio [1,2,3,4,5]. Titanium and its alloys have several advantages and, therefore, have been recognized as potential materials for applications in the automotive, biomedical, and aerospace industries [6,7,8,9,10,11,12,13]. However, titanium is expensive, and—when manufacturing aerospace parts, in particular—after the conventional machining process, up to 90% of the material is discarded as chips. The ratio of raw material weight to final part weight, known as the buy-to-fly (BTF) ratio, increases. In addition, a large amount of heat is generated during the machining process, which shortens tool life and lowers dimensional accuracy. Hence, several studies have been conducted to replace such machining processes and improve the BTF ratio.

Titanium has a low formability at room temperature owing to its crystalline structure. Hexagonal close-packed (HCP) structures are more difficult to deform than other crystal structures, such as face-centered cubic or body-centered cubic structures, because the structure has fewer slip systems. The Ti-6Al-4V alloy sheets are dominated by the α phase of the HCP structure at room temperature and have low formability due to the high critical resolved shear stress of the non-basal slip system. Materials such as the Ti-6Al-4V alloy with fine crystal grains exhibit superplastic behavior at high temperatures in the range of 800–900 °C over 0.5 T_m_, where T_m_ is the melting point in Kelvin, and at a low strain rate of 10^−3^/s or lower. During this process, the grain boundaries slide, thereby resulting in an elongation that is hundreds of times higher than that of the material at room temperature.

Diffusion bonding (DB) is a solid-state technology during which metals are exposed to temperatures below their melting points to induce bonding via atomic diffusion between two surfaces [14,15,16]. The roughness of the metal surfaces in contact is an important factor in DB for sharing free electrons sufficiently between these surfaces. Generally, DB is conducted under high-temperature and high-pressure conditions in an inert gas atmosphere. The temperature range used in this method is 50–75% of the melting point of the metal.

Superplastic forming (SPF) is a well-known application that is combined with DB for applications in titanium alloys, such as Ti-6Al-4V and TA7 [17,18,19,20,21,22]. SPF uses inert air to form parts or specimens in a warm environment. This method is also called pneumatic or blow forming [23,24,25,26]. Furthermore, SPF has several applications. Hybrid forming processes combining SPF with other forming methods, such as a two-step hybrid process comprising draw forming followed by consecutive SPF, were developed to reduce process time or to prevent failure and complete the final product of the part [27,28].

**Figure 1:**
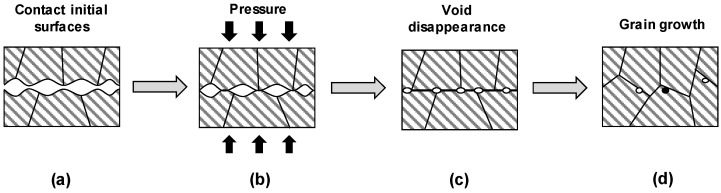
Schematic of the diffusion bonding process (**a**) contact of two independent surfaces, (**b**) application of pressure from either side, (**c**) disappearance of void, and (**d**) grain growth with grain boundary movement.

A typical DB process is illustrated in Figure 1. As shown in Figure 1a, two independent surfaces are in contact, and the materials may be similar or dissimilar [29]. To improve bonding, each surface can be polished, as surface roughness affects bonding quality [30]. Subsequently, pressure is applied from either side. The pressure usually originates from the SPF process that Rockwell developed in 1973 [31]. Owing to pressure, the overall spacing between the surfaces almost disappears, and the gaps that did not disappear remain as voids, as shown in Figure 1b,c. Finally, the grain boundaries move actively, and the voids almost disappear. Recrystallization often occurs during this process, and various surfaces or oxide films are generally dissolved in the base material, as shown in Figure 1d.

Although using SPF to generate pressure has several advantages, building equipment using this method is difficult, and forming specific shapes requires additional processes. These limitations motivated the introduction of a bending process in addition to DB in this study. When the bending process is introduced, not only can the press equipment be used without SPF equipment, but it is also possible to escape from the shape limitations that the SPF/DB process has [32]. Through the bending process instead of SPF, a more flexible shape can be achieved. Although previous studies adopted a bending process with DB to manufacture titanium alloys such as Ti22Al25Nb, each process was applied sequentially [33,34,35].

In this study, however, DB and the bending process were applied simultaneously to bond two independent metals. This process was named the one-step hybrid bending/diffusion bonding process (HB/DBP). The BTF ratio was expected to be minimized by applying the HB/DBP. The bonding characteristics of titanium-alloy sheets were estimated and analyzed. Microstructural analyses using optical microscopy (OM) and scanning electron microscopy (SEM) equipped with electron backscatter diffraction (EBSD) were used to determine the quality of the bonded product. A lap shear test was performed to evaluate the shear strengths of the bonded specimens according to the HB/DBP conditions. Creep behavior was analyzed using numerical simulations, and the failure phenomena was also discussed. In the numerical approach, viscoplastic or time-dependent material properties were characterized at high temperatures. The potential of the HB/DBP process in producing aircraft parts and the effect of viscoplastic behavior on the reliability of the final product were discussed in this study.

## 2. Materials and Methods

### 2.1. Materials

**Figure 2:**
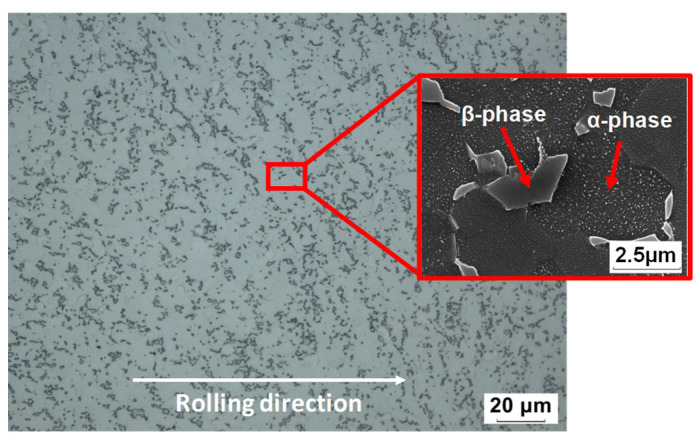
Microstructural image of the as-received Ti-6Al-4V sheets.

The material used in this study was a 2.032 mm thick mill-annealed Ti-6Al-4V alloy sheet (made by Howmet Aerospace Inc., Pittsburgh, PA, USA). The optical and scanning electron microscopic images of the as-received Ti-6Al-4V sheets are shown in Figure 2. The chemical composition of the material is listed in Table 1.

Aluminum and vanadium are present in amounts of 6.75 and 4.5 wt.%, respectively, in this alloy. However, other elements can be added to change the transition temperature from α phase to β. The properties and microstructure of the Ti-6Al-4V alloy can be varied by varying the heat-treatment history and oxygen content. The typical mechanical properties of the as-received Ti-6Al-4V at room temperature are summarized in Table 2. E, $\sigma_{YS}$, $\sigma_{TS}$, $e_{U}$, and $e_{T}$ are the elastic modulus, the yield stress, the (ultimate) tensile stress, the elongation until yield stress, and the elongation until (ultimate) tensile stress, respectively.

### 2.2. Tensile Properties

**Figure 3:**
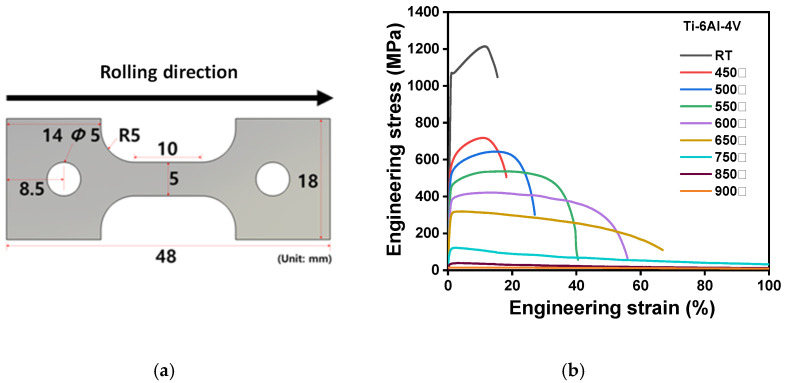
(**a**) Geometry of the specimens used for the uniaxial tensile test. (**b**) Engineering stress and strain curves with respect to temperature. R5 and RT abbreviate 5 mm radius and room temperature, respectively.

To measure the mechanical properties of the titanium alloy, subsize designed specimens of the ASTM-E8 standard [36] are prepared along the rolling direction, as shown in Figure 3a. Specimens of 2.032 mm thickness are used for the uniaxial tensile tests. Prior to the tensile tests, an HB/DBP was simulated to determine the strain-rate range. During this simulation, the maximum strain rate of the critical elements was 10^−3^/s. Therefore, the developed forming process was assumed to have a quasi-static status and 10^−3^/s was used as the strain rate.

A series of uniaxial tensile tests with respect to temperature and strain rate was performed using a universal tensile tester with a maximum load capacity of 10^4^ kg. The engineering stress–strain curves with respect to temperature are shown in Figure 3b. Voce-type hardening laws were used concerning the temperatures, as follows:

(1) $$\bar{\sigma }=A+B\left\{1-\exp(-C\bar{\varepsilon })\right\},$$

where $\bar{\sigma }$ and $\bar{\varepsilon }$ are the effective stress and strain, respectively. Additionally, *A*, *B*, and *C* are material parameters of the Voce-type hardening law. These parameters with respect to temperature are listed in Table 3. Calibration was performed based on a quasi-static strain-rate condition of 10^−3^/s.

### 2.3. Visco-Plastic Properties

**Figure 4:**
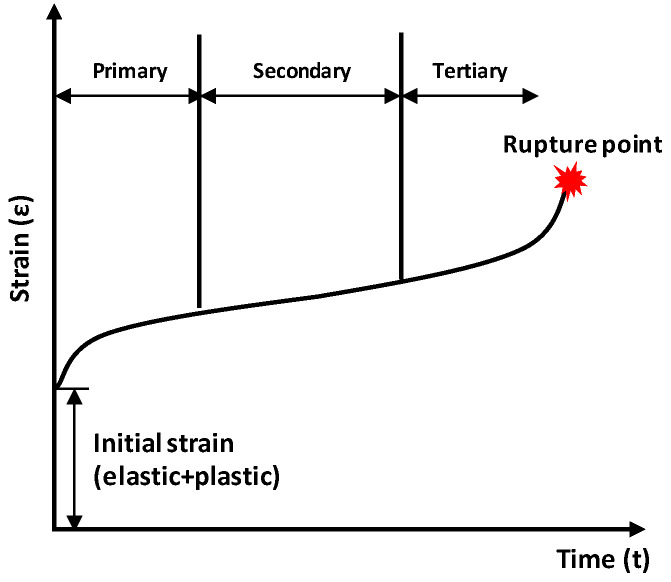
Characteristic curve showing the general creep behavior of metals [38].

Viscoplasticity, which is a time-dependent mechanical behavior, is the deformation response that depends on the duration of an applied force. These properties are normally observed in viscous materials such as polymers. Metals tend to exhibit time-dependent superplastic behaviors as the temperature increases. Creep tests, in which the change in deformation is measured over time under constant stress, are a well-known method for obtaining viscoplastic properties. The general creep behavior of metals is shown in Figure 4. After the initial accelerated primary region, the deformation reaches a stable state, which is known as the secondary region. When the limit point is reached, deformation occurs rapidly, eventually reaching the rupture point. As the tertiary region tends to end quickly, characterizing the primary and secondary regions is important using the creep test. The Norton–Bailey-based constitutive law expressed in Equation (2) is used to represent the primary and secondary regions of creep behavior.

(2) $$\dot{\bar{\varepsilon }}=\dot{\bar{\varepsilon }}(t,\bar{\sigma })={\dot{\bar{\varepsilon }}}_{0}{\left(\frac{\bar{\sigma }}{{\bar{\sigma }}_{0}}\right)}^{n}t^{m},$$

where $\bar{\sigma }$, $\dot{\bar{\varepsilon }}$, and t are the effective stress, effective strain rate, and time, respectively. Additionally, ${\dot{\bar{\varepsilon }}}_{0}$, n, and m are material parameters varied depending on temperature, and ${\bar{\sigma }}_{0}$ is the reference equivalent stress. The constraint conditions are −1<m≤0 and that ${\dot{\bar{\varepsilon }}}_{0}$ and n must have positive values. Despite being popular, detailed descriptions in the literature of the characterization method using the Norton–Bailey-based constitutive law are limited, and the discussions on necessary test conditions and methods for determining physical properties are not specifically referred to [37]. Therefore, the explicit characterization method is discussed in this section.

**Figure 5:**
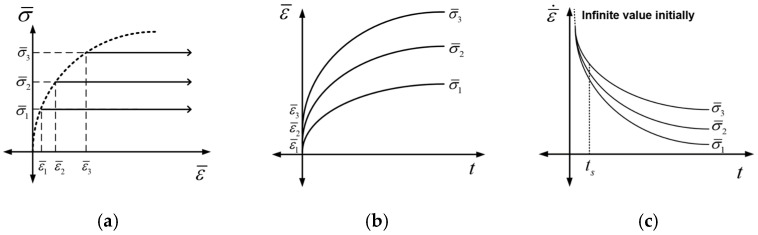
Schematic of three creep tests in the (**a**) effective stress and effective stain domain, (**b**) effective strain and time domain, and (**c**) effective strain rate and time domain.

Three tests are recommended to determine the material parameters included in Equation (1). The dependence of the viscoplastic behavior on the test conditions is shown in Figure 5. Three stress conditions with different magnitude (${\bar{\sigma }}_{1}<{\bar{\sigma }}_{2}<{\bar{\sigma }}_{3}$) are selected with some degree of stress for the uniaxial tensile test at a target temperature, as shown in Figure 5a. The strain increases with time, and each condition yields a different initial elastoplastic strain (${\bar{\varepsilon }}_{1}<{\bar{\varepsilon }}_{2}<{\bar{\varepsilon }}_{3}$) before the start of the primary region, as shown in Figure 5b. During the test, the strain rate under each condition converges to a specific value in the secondary region, as shown in Figure 5c. As the initial strain rate is conceptually infinite, a stable time ($t_{s}$) must be determined to establish a criterion that shows the differences between the three conditions.

**Figure 6:**
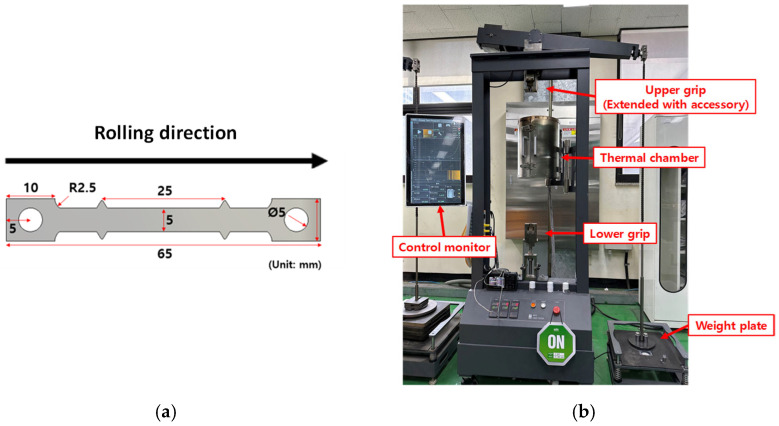
(**a**) Geometry of the specimens used for the creep test and (**b**) apparatus used for creep test at high temperatures.

**Figure 7:**
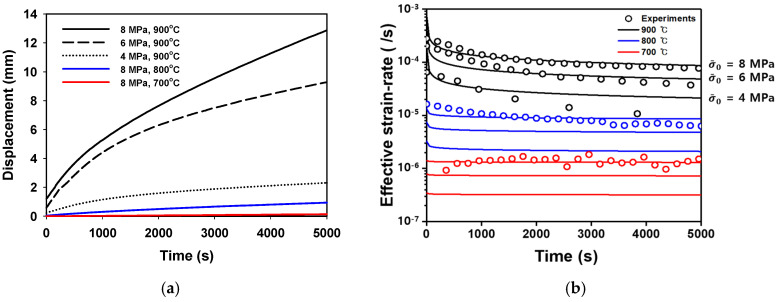
(**a**) Experimental results obtained using each condition in the creep tests, and (**b**) calibrated viscoplastic model for each condition (the solid lines follow the creep model in Equation (2)).

The maximum pressure used in the HB/DBP was 8 MPa; therefore, 8 MPa was set as the upper limit of the creep test for determining viscoplastic behavior. For determining stress-dependent behavior, 8, 6, and 4 MPa were applied to the designed specimen shown in Figure 6a using a creep tester machine (ARTE model, MTDI Inc., Jinju, Republic of Korea), as shown in Figure 6b. For temperature sensitivity, creep tests with 8 MPa at 700 °C, 800 °C, and 900 °C were also performed, as shown in Figure 7.

The small viscoplastic deformation observed at 700 °C was used as the lower bound of the model. This indicates that pressures lower than 8 MPa can result in weak viscoplastic behavior owing to small deformations, even at the highest load condition. Moreover, 8 MPa was used as the reference equivalent stress (${\bar{\sigma }}_{0}$). Each temperature was assumed to yield time-dependent behavior related to stress. The n-stress value was commonly used. Owing to the dynamic effect in the initial stage, the stable time ($t_{s}$) mentioned in the previous section was 1000 s. The material parameters in Equation (2) with respect to temperature are summarized in Table 4.

### 2.4. HB/DBP Process

**Figure 8:**
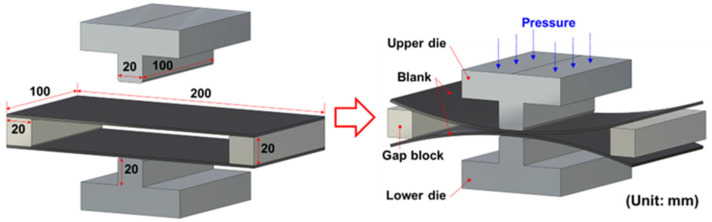
Schematic of the HB/DBP process.

Two specimens with dimensions of 200 mm × 100 mm were prepared. To remove any contaminants, the specimen surfaces were polished to a roughness less than 0.05 μm (Ra) and cleaned using ultrasonic vibration in a bath filled with >99% pure ethyl alcohol for 10 min. Owing to the nature of solid-state bonding, surface oxidation should be prevented before HB/DBP. A schematic of the HB/DBP process is shown in Figure 8. To ensure clearance between the upper and lower specimens for bending, two Inconel 718 blocks with dimensions of 20 mm × 20 mm × 100 mm were placed at both ends of the specimens.

**Figure 9:**
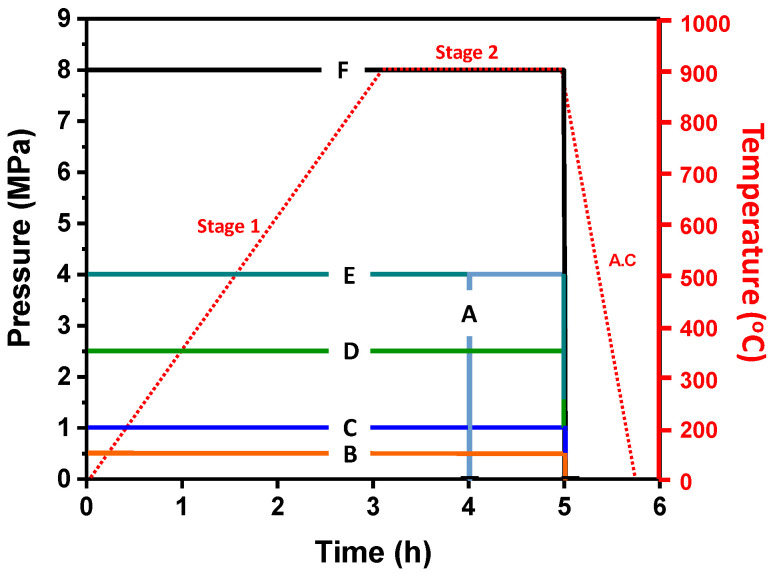
Experimental pressure conditions depending on the temperature history during the HB/DBP.

**Figure 10:**
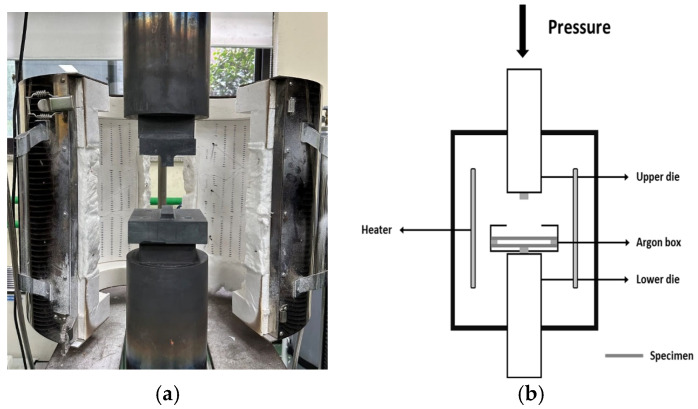
(**a**) Photograph and (**b**) schematic of the HB/DBP apparatus.

The temperature conditions of the HB/DBP are below 900 °C. Additionally, 900 °C is slightly higher than 0.5 T_m_ and lower than the phase transformation temperature at which superplastic behavior usually occurs [39,40,41]. The experimental pressure conditions with respect to the temperature history during the HB/DBP are shown in Figure 9. The temperature within a resistance-heating-type electric furnace was increased to the target temperature of 900 °C within 3 h. The temperature increase rate was set at 5 °C/min (stage 1) and then maintained for 2 h after the temperature reached 900 °C (stage 2). The oven was then air-cooled (A.C). During the temperature history, six pressure conditions were applied: A (4 MPa), B (0.5 MPa), C (1 MPa), D (2.5 MPa), E (4 MPa), and F (8 MPa). To prevent the formation of an oxide layer, the HB/DBP was performed in an argon atmosphere using a universal tensile machine with a maximum capacity of 10^4^ kg. The apparatus was composed of upper and lower dies and a heating furnace. The apparatus and schematics are shown in Figure 10.

### 2.5. Numerical Modeling of HB/DBP

To predict the creep deformation at different temperatures, the Norton–Bailey-based constitutive law expressed in Equation (2), which is a time-dependent constitutive law, is used for simplicity. The prediction was conducted using ABAQUS 2023 (Dassault Systemes). To apply the creep material properties, a visco/implicit procedure must be considered. A time–power law model equipped with a creep material was used [42]. However, the rate sensitivity term was incorrectly described in the ABAQUS 2023 manual as follows:

(3) $$\dot{\bar{\varepsilon }}=\dot{\bar{\varepsilon }}(t,\bar{\sigma })={\dot{\bar{\varepsilon }}}_{0}{\left(\frac{\bar{\sigma }}{{\bar{\sigma }}_{0}}\right)}^{n}({\dot{\bar{\varepsilon }}}_{0}t^{m}\;)$$

**Figure 11:**
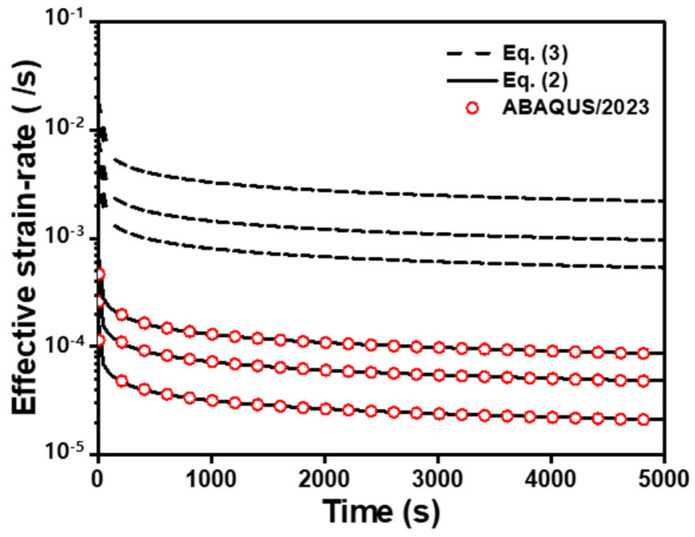
Comparison between the ABAQUS 2023 built-in creep model and Equations (2) and (3) at 900 °C.

The material parameters at 900 °C, which are summarized in Table 4, are used as the coefficients in Equations (2) and (3). The built-in model agrees well with Equation (2) as shown in Figure 11. Furthermore, Equation (2) was used to represent the creep material behavior in the ABAQUS 2023 built-in model.

**Figure 12:**
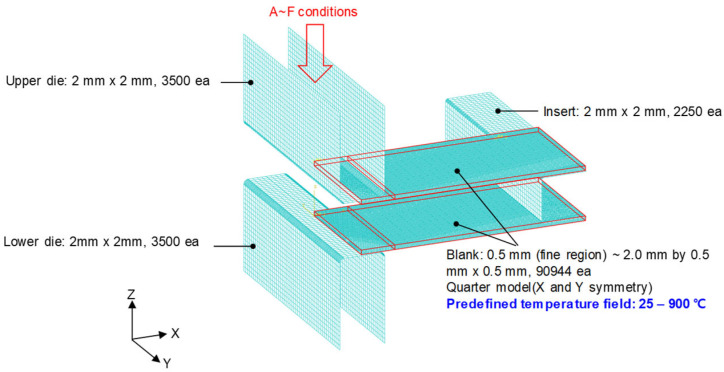
Schematic of the finite element model and constraints applied.

**Figure 13:**
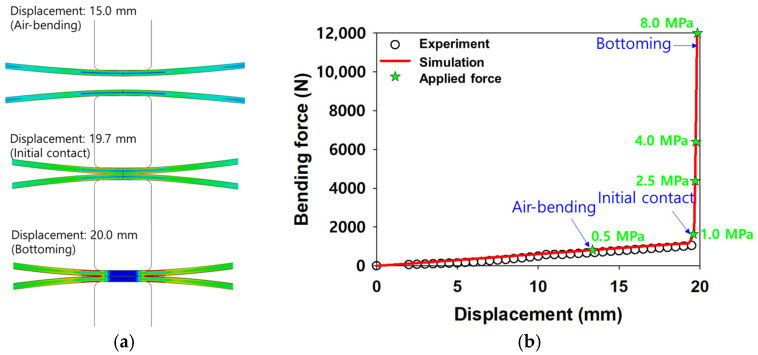
(**a**) Schematic of the air-bending, initial contact, and bottoming processes and (**b**) the resulting force versus displacement curve.

ABAQUS/Visco (implicit) was used to analyze the HB/DBP behavior. A schematic of the finite element model and the constraints applied are shown in Figure 12. Three rigid bodies, i.e., an upper die, a lower die, and an insert, and two deformable bodies, i.e., upper and lower blanks, were used in the model. A fully integrated hexahedral element (C3D8) was used as the deformable blank sheets. Four pressure conditions, i.e., 1.0, 2.5, 4.0, and 8.0 MPa, were applied. The pressures were calculated based on the initial cross-sectional area of the joint surface. The friction coefficient between the tools and specimens was assumed to be 0.2 using the general contact method. Bending occurred over 100 s, followed by a constant stress condition or creep for 18,000 s. Figure 13a shows the air-bending, initial contact, and bottoming processes in the HB/DBP. The force and displacement curves obtained from these processes are shown in Figure 13b. After the initial contact between the two specimens, the force was suddenly increased until the bottoming process occurred.

**Figure 14:**
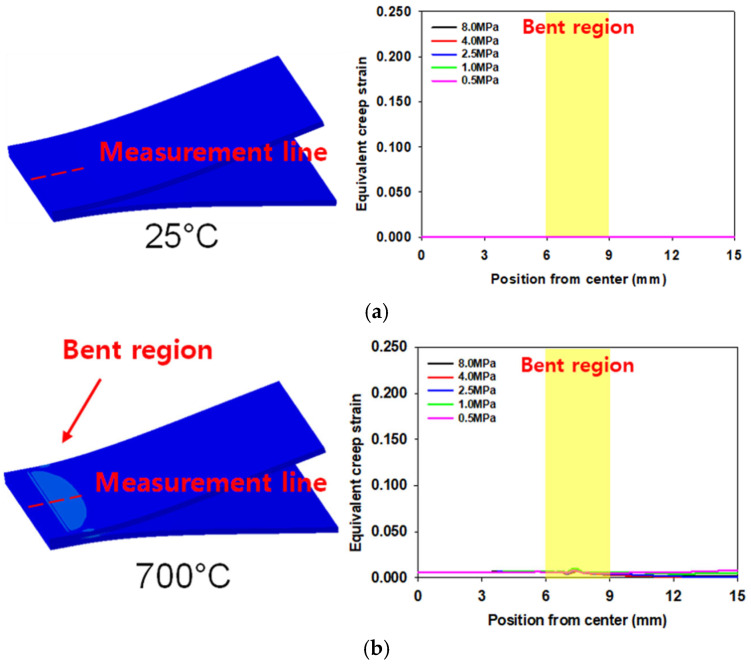

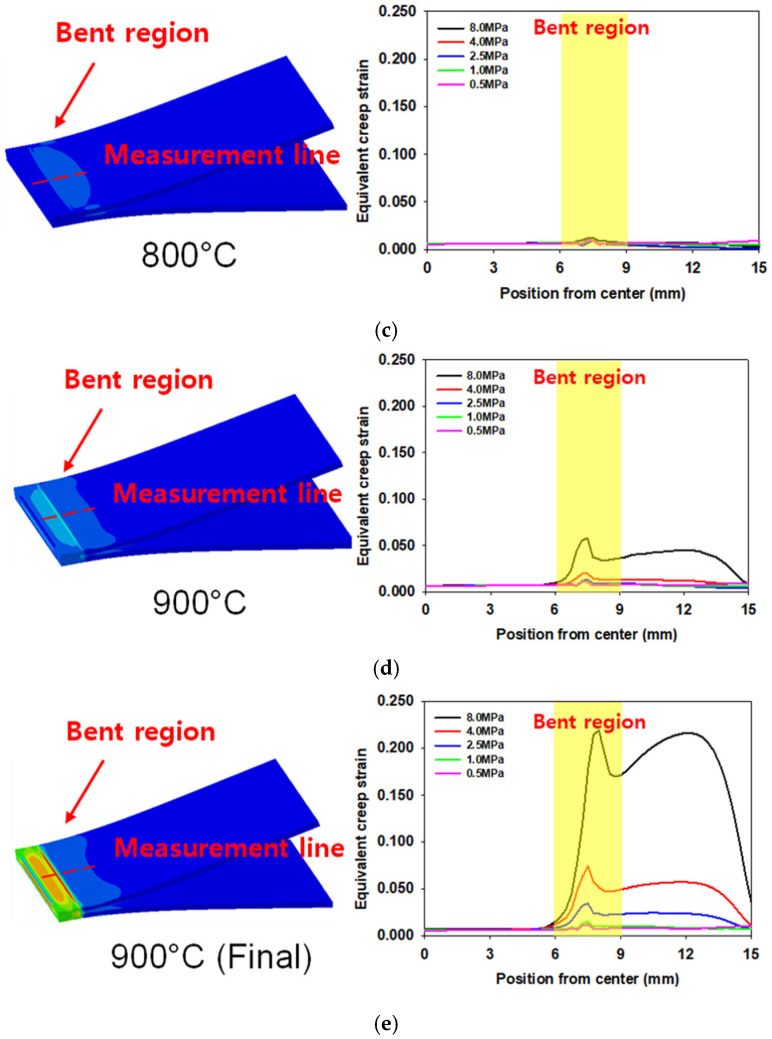
Equivalent creep strain distribution depending on temperatures (4.0 MPa is used as an example): (**a**) 25 °C, (**b**) 700 °C, (**c**) 800 °C, (**d**) 900 °C, and (**e**) 900 °C as the last step.

Figure 14 shows the equivalent creep strain distribution along the bonding section with respect to temperature. The temperature increases over time according to the profile shown in Figure 9. Figure 14d shows the equivalent creep strain distribution immediately after the target temperature of 900 °C is reached, and Figure 14e shows the results of the final experiment, i.e., after 18,000 s. No creep strain was present at 25 °C, and creep strain began accumulating as temperature increases. Creep deformation was concentrated in the bent region. Owing to the deformation caused by creep after contact, the possibility of failure in the peripheral localized area increased under conditions D–F. Among the conditions, condition F (8.0 MPa) has the most excessive equivalent creep strain distribution in the bent region. However, under conditions B (0.5 MPa) and C (1.0 MPa), the equivalent creep strains were uniformly distributed at the end of the process. Therefore, the fracture location could be determined based on the bonding strength under conditions B and C.

## 3. Results and Discussion

### 3.1. Joint Strength Resulting from the HB/DBP

**Figure 15:**
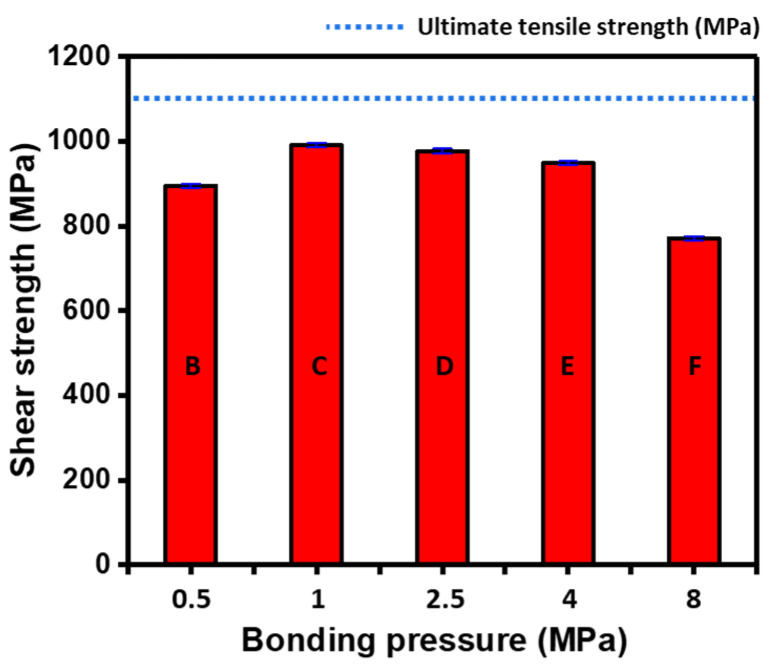
Shear strength of the joints with respect to the bonding pressure with small error.

**Figure 16:**
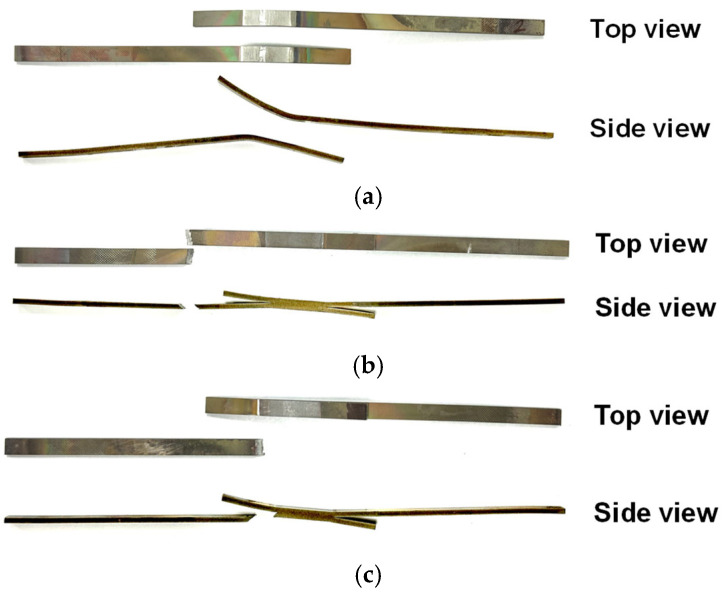
Fractured specimens under conditions (**a**) B, (**b**) C, (**c**) D, E, and F.

After the HB/DBP, a lap shear test was performed for estimating joint strength using a universal tensile tester. The total length, overlapping length, and thickness of the joint were approximately 110, 20, and 6 mm, respectively. The joint strengths under each pressure condition are listed in Table 5. Shear tests were conducted to determine whether the mechanical strength of the bonded joint was similar to that of the base material. Figure 15 shows the shear strength of the joints as a function of the bonding pressure. Figure 16 shows the fractured specimens, except for condition A, under which the joints were not bonded to each other. The strength of the base material is approximately 1050 MPa, as indicated by the blue dotted line. The shear strengths of the bonded joints were lower than that of the base material. Condition B (0.5 MPa) resulted in a relatively low shear strength of 895 MPa, and a fracture occurred at the joint as shown in Figure 16a. Under condition C (991 MPa), the shear strength of the bonded joint was slightly lower than that of the base material. A fracture occurred in the base material and not in the joints between the specimens as shown in Figure 16b. The shear strength of the base material decreased owing to the recovery phenomenon at high temperatures over a long duration. Under conditions D–F, the shear strengths decreased with the increase in applied pressure, with shear strengths of 977, 949, and 771 MPa corresponding to conditions D, E, and F, respectively. A fracture occurred at the peripheral localized area as shown in Figure 16c. As the most excessive equivalent creep strain distribution in the bending region under condition F (8.0 MPa) was predicted in the previous section, the strength was the lowest among conditions D–F due to strain localization.

**Figure 17:**
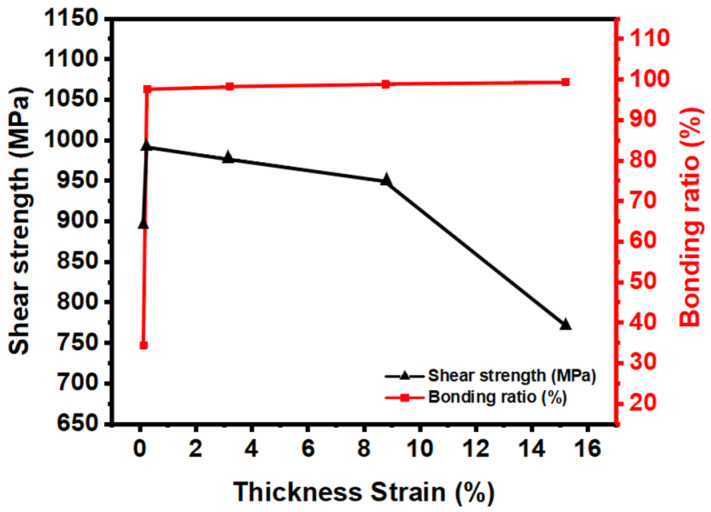
Bonding ratio, thickness strain, and shear strength under each condition.

To quantitatively determine the degree of bonding, the bonding ratio, thickness strain, and shear strength under each condition are determined and listed in Table 6 and shown in Figure 17. Thickness strain is the ratio of the reduced thickness after the HB/DBP, and the bonding ratio is the ratio of the total length of the joint interface, except for the total length of the void, which was calculated using an OM (BX53MRF-S) with an image analysis program (ZOOTOS LeopardPro) [43]. Under condition B, almost no deformation occurred throughout the thickness (0.12%), and the bonding ratio (34.44%) and shear strength (895 MPa) were relatively low. An excellent bonding ratio was obtained under conditions C–F. The required pressure was concluded to be greater than that under condition B to ensure bonding reliability. However, as the bonding ratio increased, the thickness strain increased, and the shear strength decreased. The increased pressure promoted void shrinkage at the joint interface, thus resulting in a healthy bonding ratio; however, the concentrated force applied only to the center of the sheets during the HB/DBP is too high. The concentrated pressure caused excessive creep deformation. Subsequently, the reducing thickness acted as a notch with fracture occurring early owing to reduced shear strength.

### 3.2. Microstructural Analysis

By applying pressure to each surface at room temperature, the contact of the bonding surfaces was maintained to minimize oxidation owing to the partial presence of oxygen. The high temperatures resulted in oxygen continuously diffusing into the titanium alloy surface to stabilize the β-phase-depleted region, thus forming an oxide layer known as α-case with a thickness of several microns that covered the surface of the material. This suppressed the diffusion of atoms and hindered diffusion bonding.

**Figure 18:**
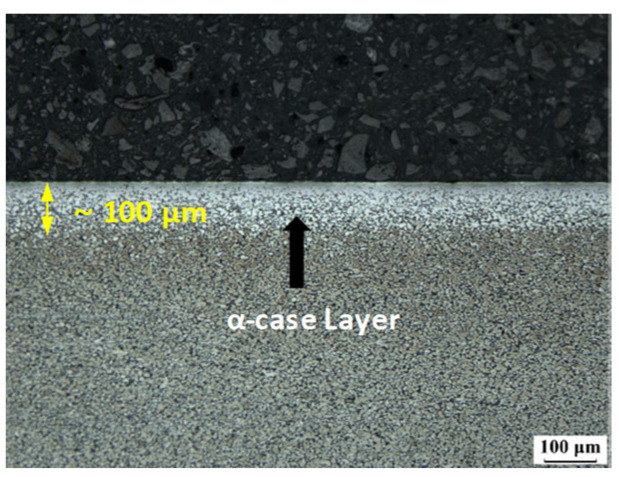
Optical image of the of α-case layer along the periphery of the cross-sectioned samples of Ti-6Al-4V.

To observe the microstructure of the bonding interface and oxide layer and determine the bonding ratio, the specimens were polished before being etched for 3–5 s using Kroll’s solution (100 mL of water + 5 mL nitric acid + 3 mL hydrofluoric acid). Subsequently, Weck’s solution (100 mL of water + 3 g of ammonium hydrogen fluoride) was used for 10 s of etching. Next, an OM, SEM energy-dispersive X-ray spectroscopy apparatus (APREO S), and EBSD (Hitachi SU6600, Tokyo, Japan) were used to characterize the microstructure and texture of, and the oxide layer on, the bonding interface after the HB/DBP. Under condition A, in which the bonding surface was exposed to a high temperature, oxygen was continuously diffused onto the surface to form an α-case layer with a thickness of approximately 100 μm, as shown in Figure 18. Interatomic diffusion was suppressed owing to the presence of this layer on the surface of the material, resulting in the failure of diffusion bonding.

**Figure 19:**
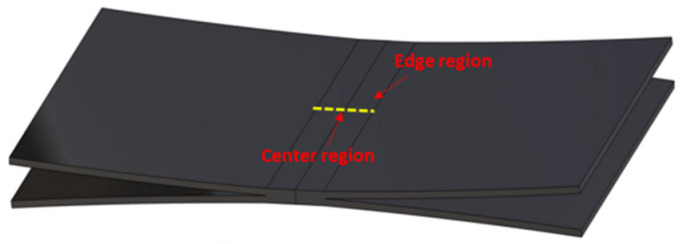
Schematic of the observation region (center/edge).

**Figure 20:**
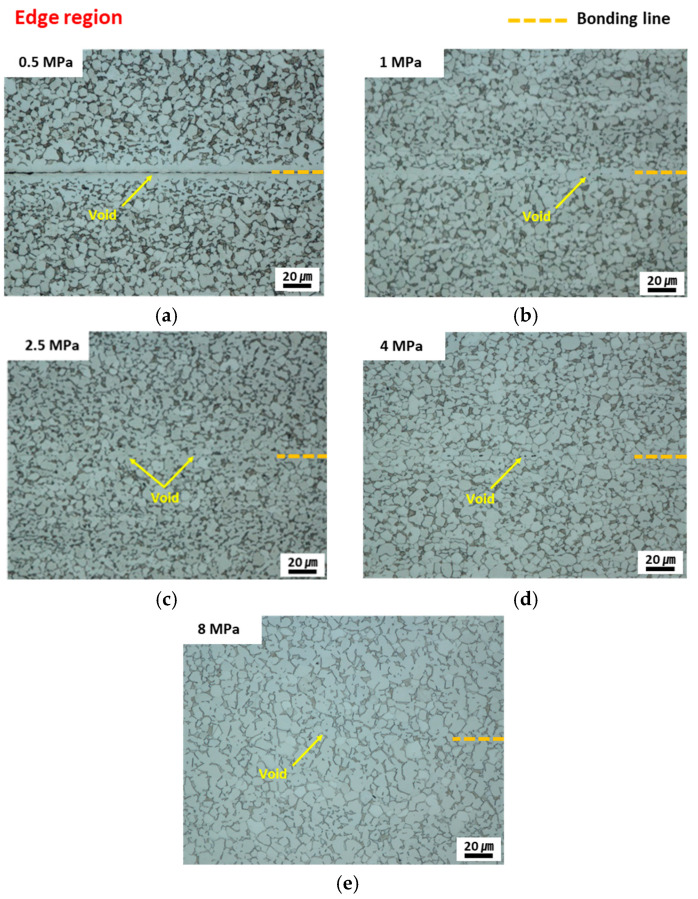
Optimal images of the edge line under conditions (**a**) B (0.5 MPa), (**b**) C (1.0 MPa), (**c**) D (2.5 MPa), (**d**) E (4.0 MPa), and (**e**) F (8.0 MPa).

To confirm the integrity of the diffusion-bonded joints under all the conditions, except for condition A under which no bonding was achieved, the microstructure of the bonding interface was analyzed, and the location of this interface is shown in Figure 19. Figure 20 shows the microstructure of the edge region. For condition B, which corresponds to relatively low applied pressure, sound bonding could not be achieved because the irregularities and voids of the bonding interface were not removed and the partially bonded area was along the centerline. As the applied pressure increased (Figure 20b–e), a continuous phase and small round pores with an increased bonding ratio were observed in the interface region in the OM analysis.

**Figure 21:**
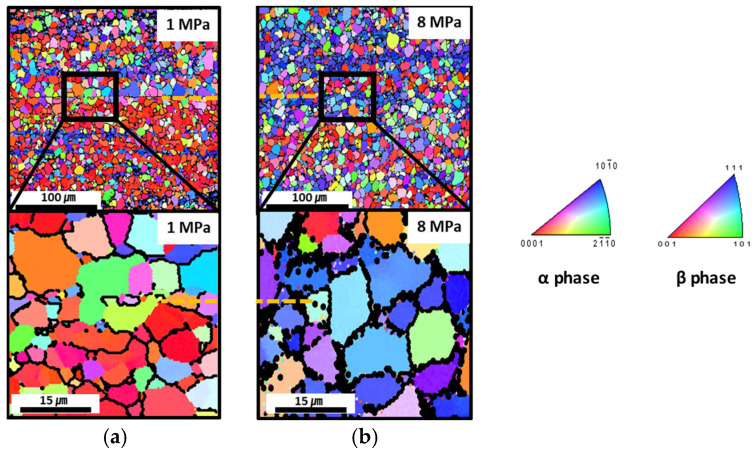
EBSD analysis and inverse pole figures along the center region for conditions (**a**) C (1 MPa) and (**b**) F (8 MPa).

An inverse pole figure map was created using EBSD analysis along the center region for conditions C and F in Figure 21. The orange dotted line represents the interface. From the enlarged image obtained under condition C, each grain orientation was confirmed to be partially connected, but the orientations of the microstructures were generally different. Therefore, the bonding was imperfect under relatively low-pressure conditions, as shown in Figure 21a. For condition F, however, interface lines and continuous grain orientations were present, showing connected grain boundaries, as shown in Figure 21b.

### 3.3. Prototype Manufacturing

**Figure 22:**
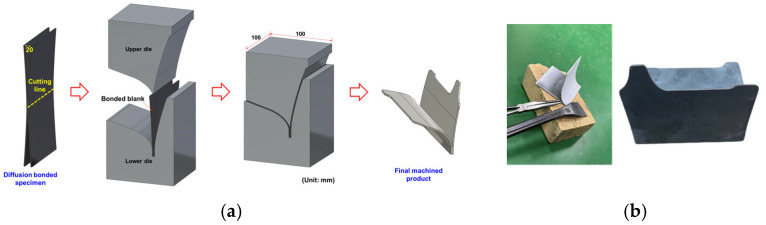
Schematic of (**a**) the gap-opening process after the HB/DBP and (**b**) the final shape of the prototype.

**Figure 23:**
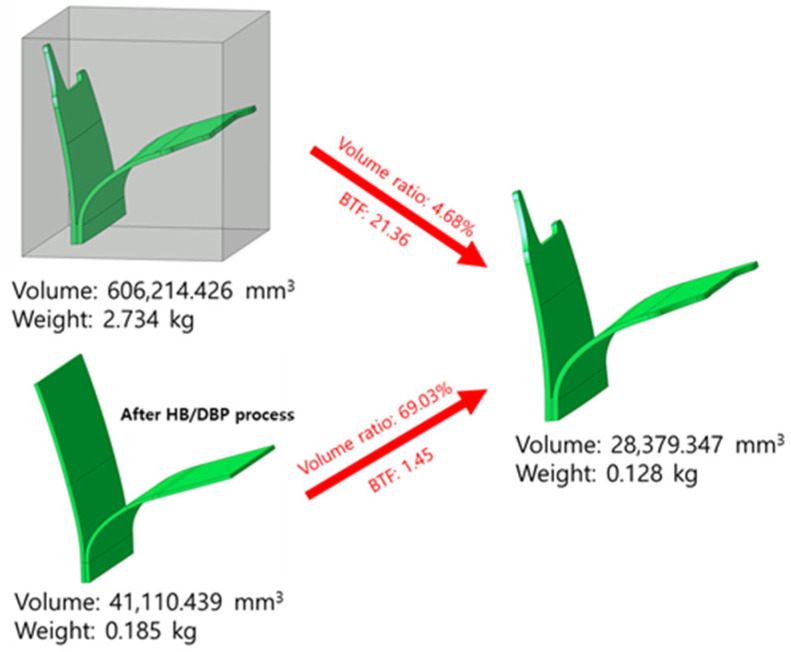
Volume and BTF ratios after the HB/DBP in comparison with those of the conventional machining process.

A prototype of a Y-shaped heat shield was manufactured using a post-gap opening process after the HB/DBP. Such a shield is mounted under the wings of an aircraft to protect the underside of these wings from the high-temperature gas injected from the engine as a part of the aerospace pylon. A schematic view of the gap-opening process and the final shape of the prototype are shown in Figure 22. After the HB/DBP was completed, the center of the specimen was cut before the end of the specimen was opened. Finally, a prototype was manufactured using a wire-cutting method. As shown in Figure 23, the volume and BTF ratios were computed. The volume and BTF ratios of the conventional machining process are 4.68% and 21.36, respectively. These values are higher than those of the developed HB/DBP process (69.03% and 1.45), indicating that the amount of waste material is reduced after the HB/DBP.

## 4. Conclusions

In this study, an HB/DBP process was developed for application to Ti-6Al-4V sheets to improve the BTF in relation to that of the conventional machining process, and the bonding characteristics of the process were evaluated. To evaluate the bonding quality, microstructural analysis and mechanical lap-shear tests were conducted. The bonding ratio, thickness strain, and shear strength of the specimens were determined based on pressure and temperature histories. The conclusions of this study are as follows:

- As the pressure was insufficient during the process, early failure occurred at the joints. The microstructure also revealed a disconnected line between the interfaces. However, when sufficient pressure was applied, the grains at the interface of the microstructure were clearly connected, as observed using a microscope. Furthermore, early failure near the joint occurred under high-pressure conditions.
- To discuss this phenomenon, the viscoplastic (time-dependent) material properties were characterized and a numerical simulation was conducted. To characterize the viscoplastic properties, a series of creep tests were performed at different temperatures. Viscoplastic deformation was observed around the bending area, which is slightly away from the punch, causing weakness around the bonding under high-pressure conditions.
- The prototype of a product was produced using the HB/DBP and gap-opening processes. The BTF ratio effectively improved from 21.36 to 1.45 in relation to conventional machining. This study demonstrates the potential of the developed process in producing aircraft parts and the importance of viscoplastic behavior for the analysis of final product reliability.

## Figures and Tables

**Table 1:** Chemical composition of Ti-6Al-4V.

Elements	Ti	Al	V	C	Fe	N	O	H
Weight (%)	87.6–91	6.27	4.5	≤0.08	≤0.4	≤0.05	≤0.2	≤0.015

**Table 2:** Typical mechanical properties of the as-received Ti-6Al-4V at room temperature.

E (GPa)	$\sigma_{YS}$ (MPa)	$\sigma_{TS}$ (MPa)	$e_{U}$ (%)	$e_{T}$ (%)
113.69	1058.60 ± 1.2	1091.60 ± 2.0	9.94 ± 1.2	16.67 ± 0.5

**Table 3:** Material parameters of the Voce-type hardening law under different temperatures.

Temperature	A	B	C
**25 °C**	1060.92	326.03	6.93
**450 °C**	560.28	165.02	29.76
**500 °C**	520.82	128.75	22.11
**550 °C**	449.45	89.99	24.03
**600 °C**	369.17	50.75	46.60
**650 °C**	279.97	38.36	294.15
**750 °C**	100.17	11.32	4136.96
**850 °C**	35.91	7.12	7.13
**900 °C**	11.52	2.99	981.82

**Table 4:** Material parameters of the creep behavior expressed in Equation (2) with respect to temperature.

Temperature	${\bar{\varepsilon }}_{o}$	${\bar{\sigma }}_{0}$	n	m
**700 °C**	1.4137 × 10^−6^	8.0	2.030	−0.0093
**800 °C**	1.3238 × 10^−5^			−0.0512
**900 °C**	7.4727 × 10^−4^			−0.2532

**Table 5:** Shear strength of joints under each pressure condition.

Pressure Conditions	Shear Strength (MPa)
B (0.5 MPa)	895
C (1 MPa)	991
D (2.5 MPa)	977
E (4 MPa)	949
F (8 MPa)	771

**Table 6:** Bonding ratio, thickness strain, and shear strength under each condition.

Condition	Bonding Ratio (%)	Thickness Strain (%)	Shear Strength (MPa)
B	34.44	0.12	895
C	97.56	0.25	991
D	98.26	3.19	977
E	98.82	8.77	949
F	99.45	15.21	771

## Data Availability

Not applicable.
